# Hand soft tissue reconstruction with dorsal metacarpal artery perforator (Quaba) flap

**DOI:** 10.25122/jml-2021-0313

**Published:** 2021

**Authors:** Anca Bordianu, Floriana Irina Leoveanu

**Affiliations:** 1. Department of Plastic Surgery, Carol Davila University of Medicine and Pharmacy, Bucharest, Romania; 2. Department of Plastic Surgery, Bagdasar-Arseni Emergency Hospital, Bucharest, Romania

**Keywords:** Quaba flap, dorsal hand flap, perforator flap, hand reconstruction, hand soft tissue

## Abstract

Hand soft tissue defects after trauma injuries or tumor excision are challenging for the plastic surgeon, regardless of the patient’s age, gender, or ethnicity. Current surgical protocols suggest protecting the main arteries by using local or free perforator flaps. This article describes the use of local perforator flaps to resurface soft tissue defects with exposed tendons, nerves, arteries and/or bones to obtain the best mobility of the flexion creases without sacrificing the main artery. We present the use of the dorsal metacarpal artery perforator (Quaba) flap in two cases, showing different types of pathology that could benefit from this method. The first case is a 43-year-old male patient, known with psoriasis, who suffered a home accident with a chainsaw. The second case is a 35-year-old woman with a round, mobile skin tumor located on the volar surface of the metacarpophalangeal joint. The patients were discharged from the hospital the next day postoperative. Both patients reported a slight bulkiness of the flap without affecting the functional outcome, preserving full mobility of the fingers. The Quaba flap is a distally based perforator axial flap suitable for soft tissue defects reconstructions, safe and easy to use, with minimal donor site morbidity. Slight bulkiness could affect the aesthetics of the hand.

## Introduction

Hand soft tissue defects after trauma injuries or tumor excision pose a great challenge to the plastic surgeon, regardless of the patient’s age, gender, or ethnicity. The chosen reconstruction method should provide the best functionality, mobility, and aesthetic result [1]. Most hand soft tissue defects result from trauma, tumor excision, scar contracture release, and burns. These cases can be managed using skin grafts, local flaps, regional flaps, and free transfer flaps [1]. Current surgical protocols suggest protecting the main arteries by using local or free perforator flaps. Maintaining the main vascular supply while resurrecting tissue defects can be obtained with minimal damage, respecting the reconstruction ladder that is adapting to the newest developments in continuously changing plastic surgery [1]. When considered appropriate by the plastic surgeon, the elective reconstruction method for hand soft tissue defects is a local flap. There are several local flaps that can be considered, with similar quality (e.g., skin texture, and pilosity level, replacing like with like etc), and discrete/minimal donor site morbidity [1].

This article describes the use of a local perforator flap to resurface soft tissue defects with exposure of tendons, nerves, arteries, and/or bones and/or to obtain the best mobility of the flexion creases without sacrificing the main artery. The dorsal metacarpal artery, a thin fasciocutaneous local flap, is one of the most effective reconstructive methods for soft-tissue defects of the metacarpophalangeal joint, web space, proximal phalanx, and proximal interphalangeal joint perforator (Quaba) flap, with reliable and relatively straightforward anatomy of the pedicle [2]. While most commonly used for dorsum soft tissue defects of the proximal phalanx, it can also have good results when used for the volar side of the proximal phalanx [3].

## Case Report

**Figure 1.**
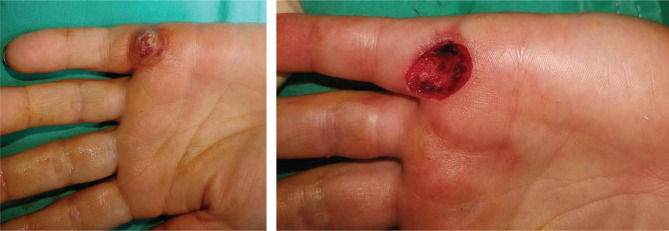
Case 2 – Before and after excision.

**Figure 2.**
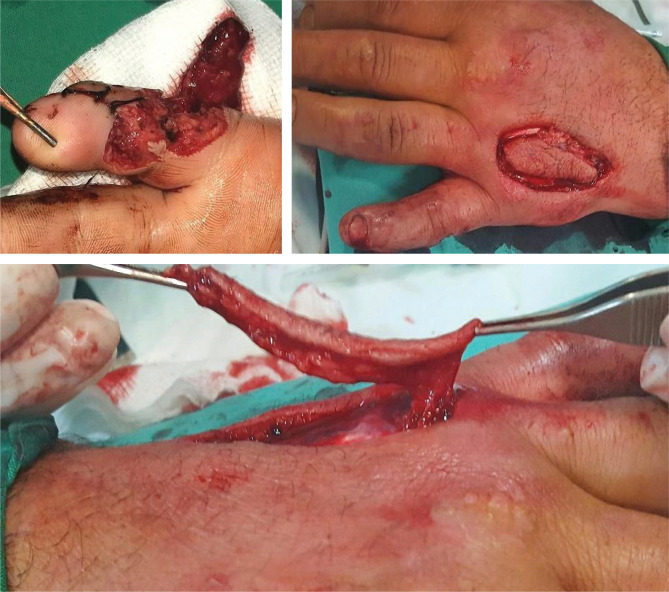
Case 1 – Defect and Quaba flap elevation.

**Figure 3.**
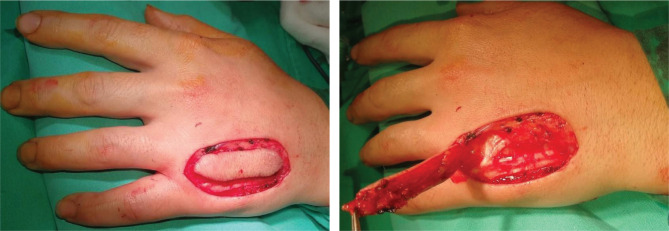
Case 2 – Quaba flap elevation.

**Figure 4.**
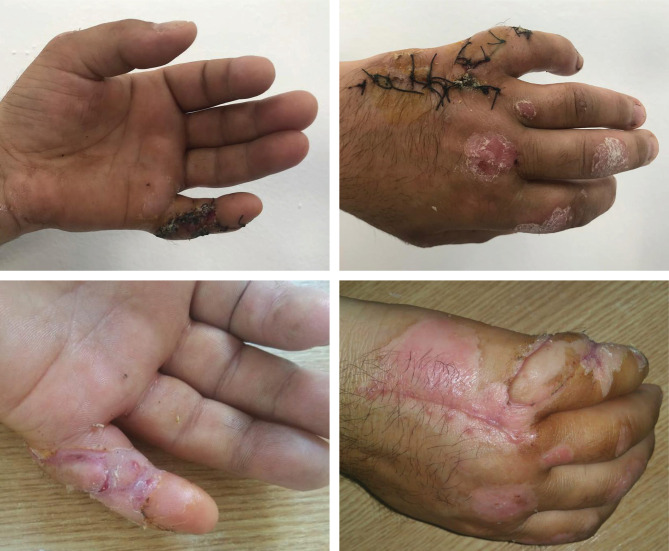
Case 1 – Postoperative aspect after 2 and 3 weeks.

**Figure 5.**
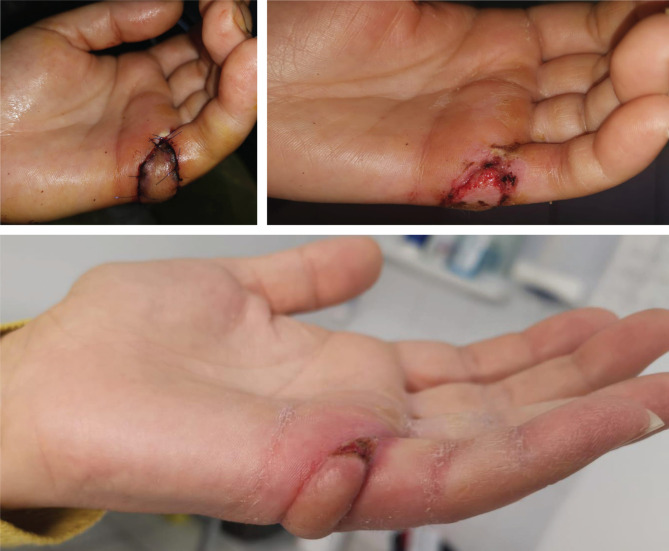
Case 2 – Postoperative aspect and after 20 days.

The Quaba flap was used in two cases, showing different types of pathology that can benefit from this reconstructive method. The first case is a 43-year-old male patient, known with psoriasis, who suffered a home accident with a chainsaw. He presented in our emergency room with a soft tissue defect of the proximal phalanx ulnar border and left-hand fifth finger proximal interphalangeal joint, with partial exposure of the flexor digitorum profundus. The 2^nd^ case is a 35-year-old woman with a round skin tumor, 1.5 cm in diameter, of brown-red color, mobile, located on the volar surface of the metacarpophalangeal joint. After the tumor excision, the underlying flexor tendons were exposed (Figure 1). Both patients were subjected to detailed medical history and physical examination, aiming to identify any prior conditions and/or scars of the affected hand dorsum that could jeopardize the flap viability. After careful debridement of the injured finger (first case) and the tumor excision with oncological safety margins (the second case), we considered a single-stage reconstructive flap surgery as mandatory, considering the location of the defect and the tendons’ exposure (Figure 1). The local perforator flap with a distally based pedicle we decided on was the Quaba flap. The Quaba flap can be elevated from the metacarpophalangeal joint to the distal edge of the extensor retinaculum, with a width between 1 and 3.5cm, centered on the intermetacarpal space [4]. It is advised not to skeletonize the pedicle due to possible venous insufficiency [2, 4]. We used the wide-awake local anesthesia no tourniquet (WALANT) technique in both cases, which provided the patient with better comfort during the surgical procedure. In addition, this type of anesthesia facilitates the safe dissection of the pedicle without the unwanted side effects associated with opiates or sedation[5]. The elevated flaps were projected on the 4^th^ intermetacarpal space, with the width of 1.5cm and the length of 4cm. The first incision was made on the radial side, dissecting the flaps superficially to the paratenon and the fascia of the dorsal interosseous muscle. The next step was raising the flaps from proximal to distal, preserving the sensory dorsal ulnar nerve up to the juncturae tendinea, as, distal to this point, the pedicle can be visualized [4, 6]. In both cases, a distal incision was performed close to the metacarpophalangeal joint for facilitating flap mobilization (Figures 2 and 3) [6]. To create a path from the flap pedicle to the defect, the skin was incised, and the flap was rotated, covering both the incision and the defect. The donor site was closed primarily with an intradermal suture for the best aesthetic result. Both patients were discharged from the hospital the next day, and they regularly returned to our hospital for follow-up check-ups. For the first case, the flap healed primarily after 14 days, without any vascular suffering or infection, with optimal coverage of the tendons. The patient fully recovered finger mobilization and function (Figure 4). For the second case, the flap suffered minor distal venous congestion, treated conservatively with dressings. The wound completely healed after 20 days (Figure 5).

**Figure 6.**
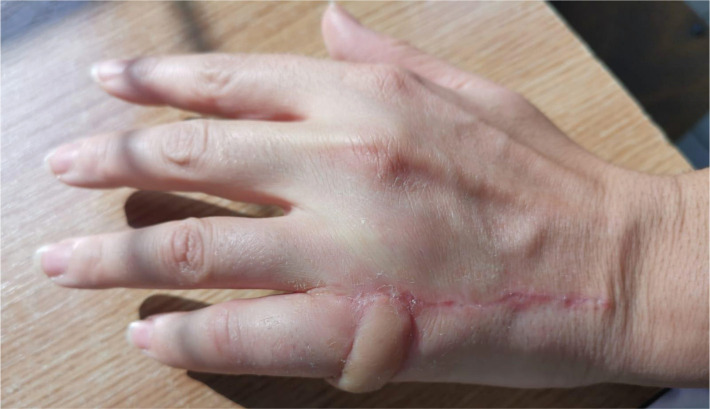
Case 2 – Postoperative aspect after 3 weeks.

In both cases, the donor site healed uneventfully, with minimal, linear scar formation (Figure 6). Both patients reported a slight bulkiness of the flap without affecting the functional outcome, preserving full mobility of the fingers (Figure 6).

## Discussion

In the surgical treatment of such defects, the main goal is to successfully cover underlying structures, such as tendons, nerves, arteries, and/or bones, and, if the case, to reconstruct the flexion creases, providing a good functional outcome. The Quaba flap is an easy-to-use local perforator flap, with minimal donor site morbidity, with a simple and straightforward rising technique [7, 8]. The palmar and the dorsal metacarpal artery system anastomoses distally, where it branches out a perforator, known as the Quaba perforator, that supplies the hand dorsum [4, 9]. As a constant palmar-dorsal small size vessel, this Quaba perforator, arising from the digital web space, is used in the flap with the same name [10]. Using vascular Doppler investigation to find the perforator is unnecessary, as the anatomy is quite constant and reliable: the perforator is located distal to juncturae tendinea, at the 2^nd^, 3^rd^, and 4^th^ interdigital space, at 0.5–1cm proximal to the metacarpophalangeal joint [2, 11].

Unlike the dorsal metacarpal artery flap, the Quaba flap is not sacrificing the dorsal metacarpal artery, a vessel that provides the main blood supply to the finger [6, 10]. While the digital artery perforator flap is also a well-known workhorse flap for hand reconstruction, it is used for fingertip defects in most cases. For the presented cases, this flap was less appropriate because of the positioning and size of the defect [12]. Another option could have been the palmar artery perforator flap. While it is known to have good results in the surgical treatment of the volar side and the little finger defects, we decided to apply the Quaba flap to avoid scarring the dominant border of the hand [13]. For Caucasians, in general, hand dorsum and the volar skin are similar in color, thickness, elasticity, and consistency, making this method very suitable for the local defect reconstruction [14–15]. A color mismatch could occur in patients with darker skin pigmentation, while pilosity issues may arise for male patients. This reconstructive method is well accepted by the patients, as it gives the possibility to primary close the donor site with low morbidity and good cosmetic results [8].

Twisting the pedicle of the flap could be a possible cause of venous congestion[16]. Also, tunneling the flap could lead to the same complication. For this reason, it is recommended to incise the skin between the donor site and the defect to minimize the risks [14]. The Quaba flap is contraindicated in patients with hand infections, as there is a higher risk of total flap loss [11].

## Conclusion

The Quaba flap has the advantage of being a distally based perforator axial flap that can reconstruct soft tissue defects. It is a safe flap, with an easy operative technique, without significant intraoperative or postoperative complications, minimal donor site morbidity, and good functional and aesthetic outcomes. Slight bulkiness could affect the aesthetics of the hand.

## Acknowledgments

### Conflict of interest

The authors declare that there is no conflict of interest.

### Consent for publication

The full responsibility for all written information in the article belongs to the authors. The patients have given consent to the authors for publishing personal information and images.

### Authorship

AB took part in the conceptualization, visualization, writing of original draft, review & editing. FIL took part in the conceptualization, visualization, and writing of the original draft.
